# An 18-Month Analysis of Bond Strength of Hot-Dip Galvanized Reinforcing Steel B500SP and S235JR+AR to Chloride Contaminated Concrete

**DOI:** 10.3390/ma14040747

**Published:** 2021-02-05

**Authors:** Mariusz Jaśniok, Jacek Kołodziej, Krzysztof Gromysz

**Affiliations:** Faculty of Civil Engineering, Silesian University of Technology, 5 Akademicka, 44-100 Gliwice, Poland; jacek.kolodziej@polsl.pl (J.K.); krzysztof.gromysz@polsl.pl (K.G.)

**Keywords:** concrete, reinforcing steel, bond strength, zinc coating, HDG, corrosion, chlorides, pullout, EIS, LPR, optical measurements, 3D scanning

## Abstract

This article describes the comparative analysis of tests on bond strength of hot-dip galvanized and black steel to concrete with and without chlorides. The bond effect was evaluated with six research methods: strength, electrochemical (measurements of potential, EIS and LPR), optical, and 3D scanning. The tests were conducted within a long period of 18 months on 48 test elements reinforced with smooth rebars ϕ8 mm from steel grade S235JR+AR and ribbed rebars ϕ8 mm and ϕ16 mm from steel grade B500SP. The main strength tests on the reinforcement bond to concrete were used to compare forces pulling out galvanized and black steel rebars from concrete. This comparative analysis was performed after 28, 180, and 540 days from the preparation of the elements. The electrochemical tests were performed to evaluate corrosion of steel rebars in concrete, particularly in chloride contaminated concrete. The behaviour of concrete elements while pulling out the rebar was observed using the system of digital cameras during the optical tests. As regards 3D scanning of ribbed rebars ϕ8 mm and ϕ16 mm, this method allowed the detailed identification of their complex geometry in terms of determining the polarization area to evaluate the corrosion rate of reinforcement in concrete. The test results indicated that the presence of zinc coating on rebars had an impact on the parameters of anchorage. In the case of ribbed rebars of 16 mm in diameter, the maximum values of adhesive stress and bond stiffness were reduced over time when compared to black steel rebars. Moreover, it was noticed that the stiffness of rebar anchorage in chloride contaminated concrete was considerably higher than in concrete without chlorides.

## 1. Introduction

The bond strength of reinforcing steel to concrete is a key element that determines the desired behaviour of a reinforced concrete composite. The effects observed at the interface between steel and concrete have been generally thoroughly investigated and clearly described [1,2,3,4,5]. However, introducing a galvanized concrete element to the reinforcement causes some disturbances at the metal-concrete interface. The standard explanation with reference to the mechanical aspects should also include electrochemical issues [6,7,8]. Three fundamental processes are generally involved in transmitting stress from the rebar to concrete. They are: chemical adhesion, friction between steel and concrete, and mechanical interaction occurring between the reinforcement and the cover [1,9]. Two first processes play an important role for bond strength between the smooth rebars. On the other hand, the mechanical interaction has a fundamental significance for the bond strength with respect to ribbed steel [10]. Thus, the bond stress for smooth steel rebars was only from one-third to one-half of stresses determined for the ribbed rebars [11].

Description of the contact between the rebar and concrete from the electrochemical point of view always requires with analysis of the bond between the metal and liquid filling concrete pores [12,13,14,15]. In the case of black steel, the highly alkaline pH of the pore solution (pH > 12.5) leads to the formation of an undesirable passive layer [16,17,18], whose presence is neglected in the analyses of the adhesion effect. Considering the hot-dip galvanized steel, the high pH of the pore solution creates a direct risk of partial dissolution of zinc coating, particularly for highly alkaline cements [19,20,21]. The products from zinc corrosion penetrate into concrete pores that have direct contact with the rebar. Additionally, the presence of chlorides in concrete, which is regarded as a corrosive factor, accelerates the destruction of zinc coating [22]. However, the limit level of chlorides is more favourable to galvanized steel than the black one [23,24,25]. It is also worth mentioning that the volume of products obtained from chlorine induced corrosion of zinc is lower compared to black steel [26]. It leads to a situation in which lower tensile stresses are noticed at the same level of chloride concentrations in the immediate vicinity of the rebar. Consequently, the formation of micro-cracks and the gradual breaking of concrete cover are slowed down [27,28,29]. All of the above electrochemical aspects of the contact between concrete and galvanized or black steel rebars have a considerable effect on the bond, particularly in the presence of chlorides [12,30].

The failure of adhesion related to friction and chemical adhesion is observed during the pull-out of the rebar from concrete under certain loading [31]. The continuity of deformations of concrete and reinforcing steel is interrupted. Thus, the so called original adhesion disappears and the working phase 1 of the anchored reinforcement is completed [9]. The value of movements in this phase was ca. 1 mm [32]. At the same time, the secondary bond is observed for the ribbed rebars. It is based on transmitting the force from the rebar through its ribs to concrete. The described effect happens because of the two following reasons: necking of the reinforcement caused by increasing stresses and exceeded local pressure of ribs to concrete. Going beyond the maximum pressure values results in the formation of diagonal cracks running from the ribs. Consequently, one small crack is running from each rib of the anchored rebar under correspondingly high stresses and at reduced deformations of the reinforcement cover [32,33]. This level of loading is known as the working phase 2. A further increase in stresses in the rebar leads to the working phase 3 of the anchored rebar. In this phase concrete is spalling under the ribbed area and slipping of the reinforcement is increasing with reference to the surrounding concrete [34]. Therefore, further loading usually causes breaking of concrete cover. So, the broken cover does not function as the closed concrete ring, but as the concrete cantilever. A certain shift of the rebar in the concrete duct corresponds to each working phase [35]. This movement is related to adhesive stress f_b_ generated on the rebar surface [1]. The value f_b_ is the highest at the end of the loading phase 3, that is, at the moment of cover cracking.

The values of individual components of stress *f*_b_ and *f*_┴_ depend on the angle at which the reaction under the ribs is induced. The value of this angle is variable and within the range of 45–80° [33], depending on the force value exerted on the rebar [36] and the anchorage section. The angle value is not affected by ribbing of reinforcing steel provided that the rib grade exceeds 45° [37]. It can be explained by the deposition of spalling concrete under the ribs which results in the formation of the area inclined at ca. 45° with reference to the axis of the reinforcement [31,34]. It should be emphasized that hot-dip galvanization usually generates a thicker layer of zinc in the ribbed area, which can slightly affect the inclination of the ribs [38]. Then, a small change in the stress values *f*_b_ can be noticed compared to the rebar without zinc coating. The component *f*_┴_ can be considered as the hydrostatic pressure acting on the concrete cover from the inside. The analyses of effects observed in the concrete cover are described in the papers, inter alia [31,39].

Many tests have been recently conducted on the effect of corrosion in reinforcement on the maximum values of adhesive stresses. The tests performed on anchored rebars of 12 mm and 16 mm in diameter indicate that bond was increasing at the beginning of the corrosion process [40]. Similar tests on anchored rebars subjected to corrosion for 17 months, which led to the development of cracks of 0.7 mm in width, did not reveal any reduced resistance of anchorage [41]. However, the tests [3] demonstrated the presence of internal cracks of 0.03–0.04 mm in width which were caused by corrosion. They were found to have a significant impact (from 44% to 54%) on reducing the resistance of bond stresses. The investigated anchorages of rebars of 12 mm in diameter indicate that the corrosion at the weight loss of the rebar at the level of 0.5–0.6% caused an increase in bond by 50–60%. On the other hand, the corrosion at the weight loss of the rebar at the level of ca. 1.5% reduced the adhesive resistance by ca. 40% [42]. The corrosion of stirrups was found to have an important effect on the reduced values of adhesive stress. Consequently, greater and adverse effects on the concrete cover were observed [43,44].

According to the comparison in the paper [6], the analyses of bond strength of galvanized steel to concrete, which have been published in recent 100 years, usually focus on short-term tests, e.g., after 28 days of concrete hardening and cover a relatively small number of specimens. However, the problem of the effect of increasing strength of concrete, and the long-term impact of chlorides on the bond of galvanized reinforcement to concrete, have still not been fully examined. Hardly any published papers on testing bond strength of hot-dip galvanized reinforcing steel grade B500SP and S235JR+AR to concrete can be found. In particular, no long-term tests have been so far conducted to analyse how the development of corrosion of galvanized and black steel rebars affected the anchorage stiffness in concrete. Therefore, the tests were also performed to broaden this knowledge.

## 2. Materials

**Figure 1:**
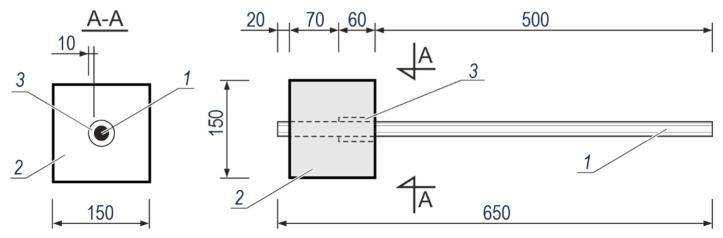
The test element used to test bond of galvanized and black steel to concrete with or without chlorides: *1*—steel rebar, *2*—concrete specimen, *3*—a 10 mm thick sleeve made of polyurethane foam with mineral filler (dimensions in millimetres).

These tests included the test elements as shown in Figure 1, which were composed of steel rebars *1* embedded in rectangular concrete blocks *2* with dimensions of 150 mm × 150 mm × 130 mm. To reduce the reinforcement contact with concrete to 70 mm in length, a polyurethane foam sleeve *3* with mineral filler was inserted at the section of 60 mm to segmentally separate steel from concrete. In total, 48 test elements were prepared from the reference concrete [45]. Concrete mix with water-cement ratio w/c = 0.45 contained cement CEM I 42.5-SR3/NA, sand, and aggregates with grains of 2–8 mm in diameter. The 1 m^3^ of the concrete mix was made up of 489 kg of cement and 1669 kg of aggregates. For 12 test elements, 3% CaCl_2_ by cement weight was added to the concrete mix to initiate corrosion. The compressive strength of concrete was determined by testing 18 cylindrical specimens with a diameter of 150 mm and a height of 300 mm. After 28 days of concrete hardening, the average strength *f*_cm_ was determined from 6 cylindrical specimens and amounted to 38.22 MPa, after 180 days—47.70 MPa, and after 540 days—50.16 MPa. The detailed results are shown in Table A1, Appendix A.

The test elements were reinforced with ribbed and smooth rebars made of steel grade B500SP and S235JR+AR, respectively. Both steel grades are classified as low-carbon steel whose chemical composition is shown in Table 1.

**Figure 2:**
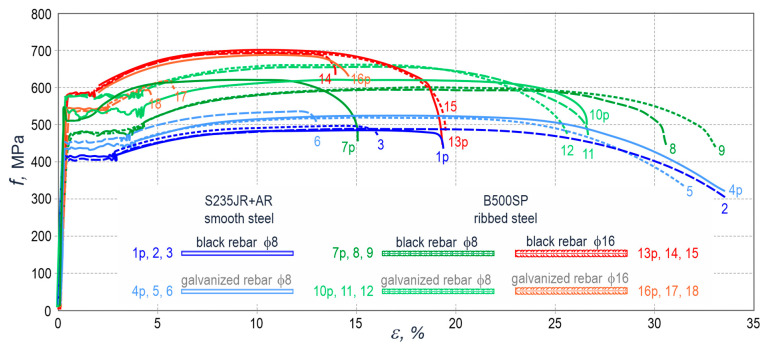
Stress *f*–strain *ε* relationship for six types of rebars tested: smooth and ribbed, galvanized, and black steel, with a diameter ϕ8 mm and ϕ16 mm, composed of two steel grades; solid lines—the rebars tested after pulling out the specimens from concrete (1p, 4p, 7p, 10p, 13p, 16p), dashed lines—the rebars that were not used in the test elements (2, 3, 5, 6, 8, 9, 11, 12, 14, 15, 17, 18).

Figure 2 presents the “stress *f*–strain *ε*” graphs for experimentally determined ductility of all types of rebars subjected to the tests. The tests were performed on 18 specimens of reinforcement, six of each steel grade and diameter. Each group of six reinforcement specimens contained two rebars that were a part of the test elements-after their pull-out from concrete. The typical yield strength *f*_yk_ for all test specimens of steel was within the range of 400–600 MPa, which is recommended by Eurocode [46]. The ductility index of this type of steel can be generally attributed to class C [46] that implies the typical strain *ε*_uk_ ≥ 7.5% corresponding to the maximum force. Numerical values for strength and tensile behaviour of rebars subjected to the tests are listed in Table A2, Appendix A. They were determined using the analysis of graphs shown in Figure 2.

The reinforcement used in the specimens was composed of 12 rebars with a diameter ϕ8 made of smooth steel S235JR+AR, 12 rebars with a diameter of ϕ8 and 24 rebars with a diameter of ϕ16—made of ribbed steel B500SP. Half of 48 rebars were galvanized in molten zinc at a temperature of ca. 450 °C prior to setting them in concrete. Zinc bath for 120 s produced a zinc coating of ca. 100 µm in thickness. The detailed analysis of the quality of the zinc coating on ribbed rebars of the same batch and made of steel grade B500SP is presented in the paper [38]. It also includes the effects of chloride-induced degradation of the coating.

## 3. Methods

**Figure 3:**
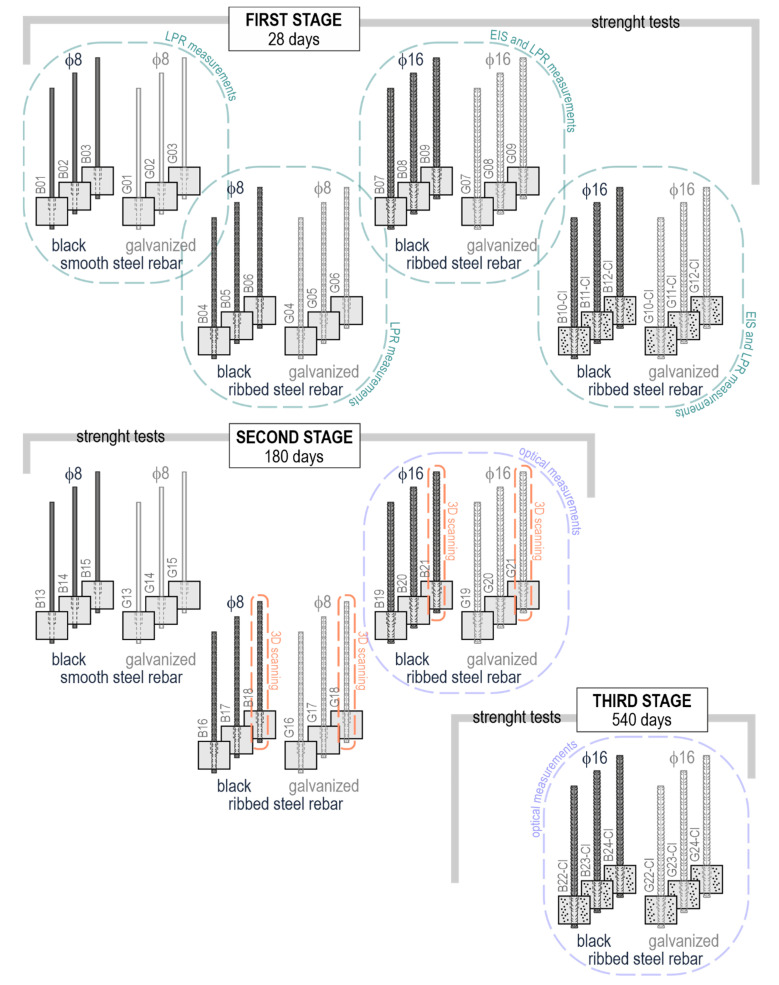
Graphical representation of three test stages of the tests conducted on different types of the test elements with five research methods.

The tests were conducted in three stages using six research methods: (1) strength tests on rebar bond to concrete, (2) optical tests and (3) 3D scanning. Moreover, three types of electrochemical tests were performed: (4) the measurements of potential, (5) Electrochemical Impedance Spectroscopy (EIS), and (6) Linear Polarization Resistance (LPR) technique. Differences in the specimens, conducted stages and the applied research methods are schematically presented in Figure 3. The specimens were numbered from 1 to 24. The independent numbering was applied to the test elements, in which black steel rebars were anchored (letter B) and to those with the galvanized rebars (letter G). The test elements with added CaCl_2_, were marked with a symbol containing Cl.

The selected ribbed rebars with a diameter of ϕ8 mm and ϕ16 mm were subjected to 3D scanning prior to the preparation of the test elements. The first stage of the tests comprised an analysis of the electrode process at the interface between reinforcing steel and concrete, which was conducted with the electrochemical methods after 3, 10, and 25 days from the preparation of the specimens. On the last day (day 28) of concrete curing in the specimens, a series of 24 measurements of the pull-out force acting on rebars began. The strength was measured for 12 test elements ϕ8 mm with smooth and ribbed rebars and 12 test elements ϕ16 mm with ribbed rebars, while six of them were embedded in concrete with chlorides. Half of the 24 rebars were hot-dip galvanized.

The second stage of the tests began 180 days (~6 months) from the preparation of the test elements. This stage included 18 elements: 12 elements ϕ8 with smooth and ribbed rebars, and six elements ϕ16 mm with ribbed rebars. All of them were placed in concrete without chlorides. Similarly to the specimens from stage 1, half of the rebars were protected with zinc coating. During the 2nd stage, the main tests on measuring the forces pulling out the rebars from concrete were performed simultaneously with the optical measurements of displacement of points painted on lateral surfaces of the specimens. Due to the limited access to the measuring equipment of the Aramis system, the optical measurements were only performed on rebars ϕ16 mm at this test stage.

The 3rd stage began 540 days (~18 months) from setting the test elements in concrete. The forces pulling out the rebars from concrete were measured in other six specimens: three of them reinforced with galvanized ribbed rebars and three others reinforced with black steel ribbed rebars, ϕ16 mm, embedded in concrete with chlorides. The optical measurements using the Aramis camera system were performed in the same way as in stage 2.

### 3.1. Strength Tests—Measuring the Force Pulling Out the Rebar from Concrete

**Figure 4:**
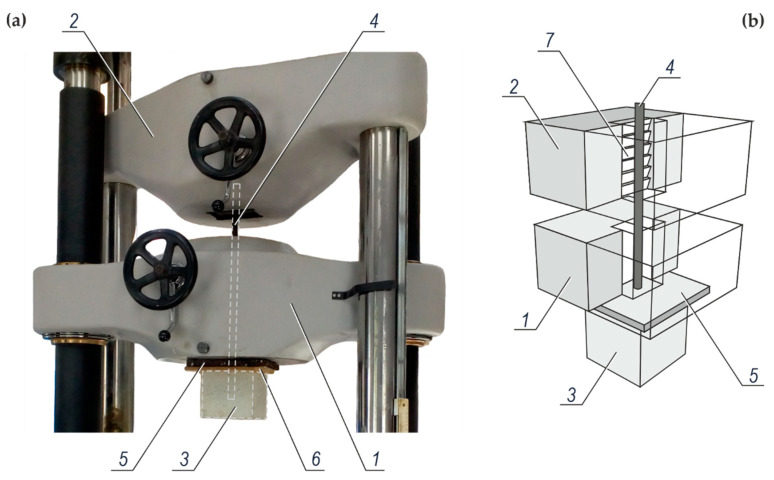
The test stand for testing the force pulling out the rebars from concrete: (**a**) view of the test machine from the Labortech company (Opava, Czech Republic) during the tests, (**b**) a diagram of the test system; *1*—a bottom holder of the test machine, *2*—a top holder of the test machine, *3*—a concrete specimen being a part of the test element, *4*—a rebar placed between holders and pulled out from the concrete specimen, *5*—steel slab transmitting force to the concrete specimen, *6*—equalizing washer made of HDF (high-density fibreboard), *7*—convergent self-clamping grips of the top holder of the test machine.

The test stand for testing the force pulling out the rebars from concrete is shown in Figure 4a, and its diagram is illustrated in Figure 4b. The test element is placed between holders of the stand.

The tests were performed with the Labortech test machine. The HDF (high-density fibreboard) equalizing washer *6* was placed on the concrete specimen *3*. Then, the steel slab *5*, transmitting uniformly the force to the concrete specimen *3*, was placed on the HDF washer. In this system the steel rebar *4* protruding from the concrete specimen passed through the bottom holder *1* of the test machine, and then was inserted into the top holder *2* with the convergent self-clamping grips *7*.

The forces pulling rebars out of concrete were measured by controlling an increment of the force over time. The force increment was constant during the 1st stage of the tests, that is, after 28 days of concrete hardening in the specimens. It was equal to 1 kN/s for a diameter ϕ16 mm and 0.5 kN/s for ϕ8 mm. Stage 2 of the tests was conducted after 180 days from the day of preparing the test elements. At this stage the force increment was increased over time. However, the measurements were interrupted when the ultimate force reached ca. 70% of the value obtained in the stage 1. The reason for that was the introduction of the Aramis system for optical measurements. The force increment was continued after changing the recording frequency of the cameras.

### 3.2. Optical Tests—Measuring Displacements of Points with the Aramis Camera System

**Figure 5:**
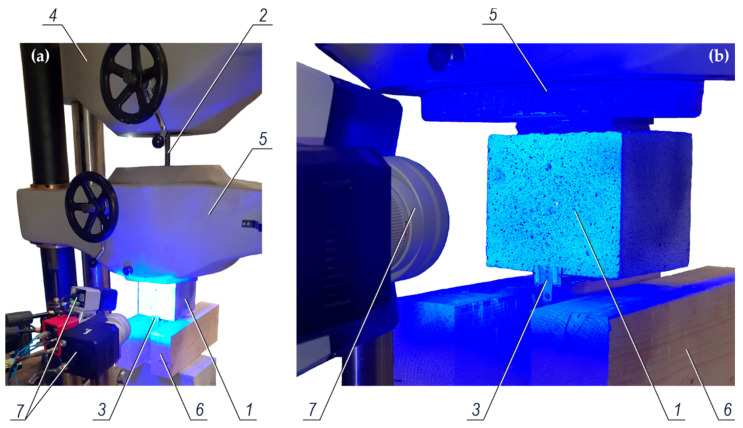
The Aramis system of cameras from the GOM company used on stage 2 and 3 of testing the force pulling out the rebar from concrete: (**a**) view of the whole test stand, (**b**) view during measurements of the concrete specimen with a spot sprayed graphite paint; *1*—concrete specimen, *2*—a steel rebar pulled out from concrete in the specimen, *3*—a bar with glued permanent measuring points, *4*—the top holder of the test machine, *5*—the bottom holder of the test machine, *6*—wood beams to support the concrete specimen after the rebar pullout, *7*—cameras recording the measurement.

The Aramis camera system from the company GOM (Braunschweig, Germany) was additionally used to provide more detailed information on the pull-out force (Figure 5a). The system was based on the Digital Image Correlation technique. The optical measurements are non-contact and are insensitive to the behaviour of materials. The system was composed of two 6 Mpx cameras (GOM, Braunschweig, Germany) equipped with 24 mm lenses to measure the test area of 120 mm × 150 mm and 90 mm in depth. The scanned area facilitates the analysis of data collected both from the whole area and the selected points. At a time before the measurements, the surface of concrete specimen *1* was painted white, and then sprayed with graphite paint (Figure 5b) to cover ca. 50% of the surface. Then, a plate *3* with glued permanent measuring points was fixed to the rebar *2*. The measurements were taken for displacements of the bar *3* with respect to the bottom edge of the concrete element, from which the rebar was pulled out.

### 3.3. Electrochemical Testing of Corrosion of Concrete Reinforcement

**Figure 6:**
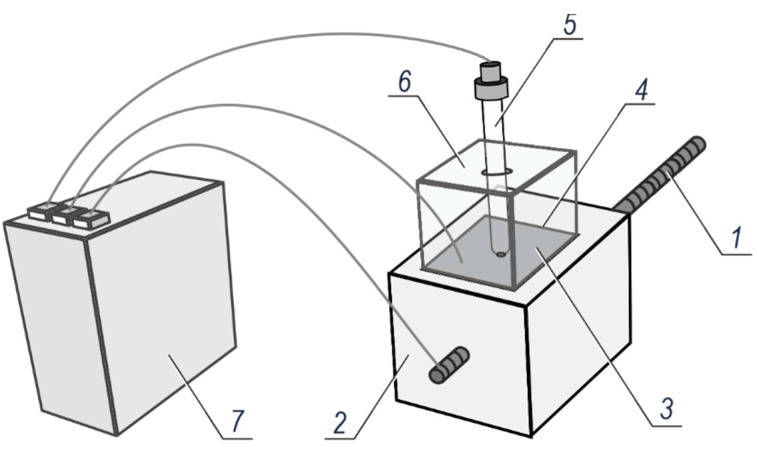
The test set-up used for the polarization tests of corrosion rate in concrete reinforcement: *1*—steel rebar, *2*—concrete specimen, *3*—counter electrode made of stainless steel sheet, *4*—felt pad, *5*—reference electrode Cl^−^/AgCl,Ag, *6*—ballast with a guide for the reference electrode, *7*—potentiostat.

The density of corrosion current was measured for the reinforcement in concrete specimens using a three-electrode arrangement illustrated in Figure 6. Both ends of the steel rebar *1* protruding from the concrete specimen *2* served as the working electrode. The counter electrode *3* made of stainless-steel sheet was placed on a wet felt pad *4*. A third electrode was the reference electrode *5* (Cl^−^/AgCl,Ag) that was stabilised in the ballast guide *6*. All three electrodes were connected to the potentiostat *7* (Gamry Instruments, Warminster, PA, USA)—the Reference 600 model from the Gamry company.

The electrochemical tests on polarization were conducted on a part of the specimens using in the first place the electrochemical impedance spectroscopy (EIS) technique. The samples were stored at ca. 20 ± 2 °C and a relative humidity of 50 ± 10%. Prior to beginning polarization tests on a given day, the top surface of the concrete sample was immersed in tap water to the depth up to 30 ± 1 mm for ca. 15 min to ensure better conductivity of concrete cover in the tested reinforcement during measurements. When the specimens were taken from water, they were connected to the potentiostat. Changes of the gradually stabilising potential were observed using the reference electrode. When changes in the potential were at the level of 0.1 mV/s, the EIS technique was used to perform the tests on the steel reinforcement in concrete. The measurements were taken in the potentiostatic mode at the fixed range of frequencies of 0.01 Hz–100 kHz and a disturbing sinusoidal signal was applied at the potential amplitude of 10 mV over the corrosion potential. After completing the impedance measurements, the changes in the potential of the reinforcing steel in concrete were again observed. The potentiodynamic measurements using the LPR technique were taken when the potential was stabilized at the level of 0.1 mV/s. The reinforcement was polarized at a rate of 1 mV/s within the defined range of potential changes from –150 mV to +50 mV against the corrosion potential.

### 3.4. 3D Scanning of Ribbed Rebars

The detailed representation of the complex geometry of ribbed rebars embedded in the concrete specimens was mapped in detail using the laser scanner Model Maker MMDx100 from the Nikon Metrology company (Leuven, Belgium). The scanning head MMDx100 based on the ESP3 technology emits a laser beam of 100 mm in width at the declared accuracy of 20 µm. Scanning of the steel ribbed rebars was supported with the 7-axis measurement arm MCAx20 from the Nikon Metrology company (Leuven, Belgium). Its measurement range is 2.0 m, the measurement accuracy of a point is 0.023 mm, and the spatial accuracy is 0.033 mm.

## 4. Results

### 4.1. Effects of 3D Scanning of Ribbed Rebars

**Figure 7:**
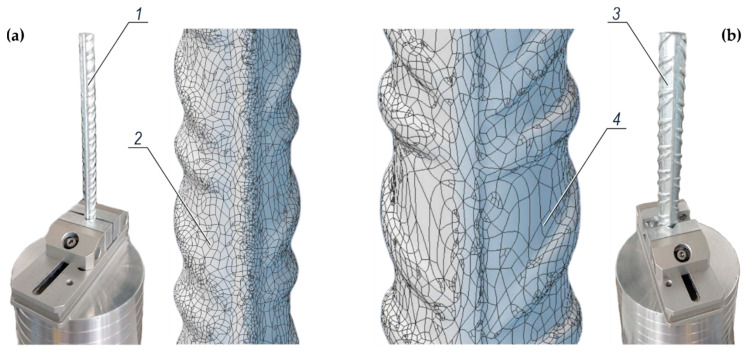
3D scanning of typical fragments of ribbed rebars made of steel grade B500SP: (**a**) a rebar with a diameter of 8 mm, (**b**) a rebar with a diameter of 16 mm; *1* and *3*—rebars ϕ8 mm and ϕ16 mm stabilised prior to 3D scanning, *2* and *4* —represented geometry of rebars ϕ8 mm and ϕ16 mm based on point cloud data.

Figure 7 present 3D images of typical fragments of ribbed rebars with a diameter ϕ8 mm and ϕ16 mm. They were scanned using the scanner (Nikon Metrology, Leuven, Belgium) specified in point 3.4. The obtained point cloud data were processed with the Focus software from the Nikon Metrology company (Leuven, Belgium). The surface of ribbed rebars was calculated using the Alibre Design software from the Datacomp company (Crakow, Poland). The calculated surface area of the ribbed rebar at the interface between reinforcing steel and the concrete specimen over a distance of 70 mm was equal to 20.10 cm^2^ for the rebar diameter ϕ8 mm, and 40.21 cm^2^ for the rebar ϕ16 mm.

The obtained detailed information on the complex geometry of typical fragments of ribbed rebars of both diameters (ϕ8 mm and ϕ16 mm) were used in described, further in this paper, tests to determine the polarized area of the reinforcement part being in contact with concrete from the specimen. This information was regarded as the required one to determine the density of corrosion current during the polarization tests using the EIS and LPR techniques.

### 4.2. Test Results for Corrosion Potential of Rebars in Concrete

**Figure 8:**
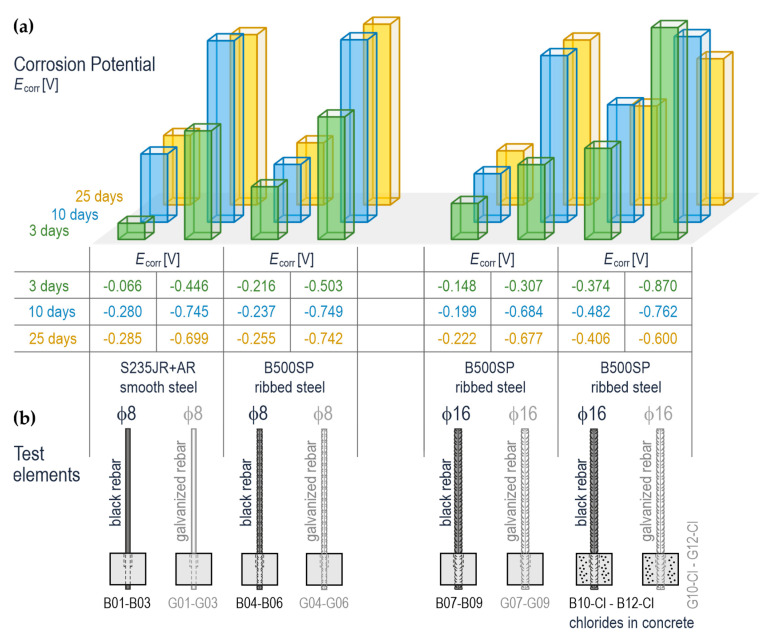
The results from electrochemical tests on reinforcing steel in concrete on days 3, 10, and 25 from setting the specimens in concrete: (**a**) corrosion potential *E*_corr_ averaging from three specimens (**b**) the test elements with galvanized and black reinforcing steel (smooth ϕ8 mm and ribbed ϕ8 and ϕ16 mm) in concrete with and without chlorides.

Figure 8 illustrates the results for the average measurements of the corrosion potential *E*_corr_ for galvanized and black reinforcing steel in 24 specimens subjected to the tests. The tests referred to the first series of the specimens, that is, B01–B12-Cl and G01–G12-Cl. The potential was measured as a preliminary element of the measurement procedure, which was required prior to the polarization tests concerning corrosion rate of reinforcement corrosion conducted with the LPR technique. The potential value *E*_corr_ was read only when its variability was stabilised at the level of 0.1 mV/s. The potential values expressed as [V] and measured against the reference electrode Cl^−^/AgCl,Ag are presented in the tabular form in Figure 8a below the bar charts.

The potential was measured each time for three specimens of the same type, and the calculated arithmetic means were inserted into the tables in Figure 8a. The measurements of *E*_corr_ were repeated three times, that is, on days 3, 10, and 25 after setting the specimens in concrete. Each of three measuring series was marked with a different colour as seen in Figure 8a to facilitate the analysis of obtained results. Moreover, the specimen sketches denoted with the rebar types and specimen symbols are illustrated in Figure 8b to connect the obtained results with particular types of specimens.

The measurement results presented in the form of bar charts demonstrate a clear difference between the potentials *E*_corr_ for galvanized and black steel. The potential of galvanized rebars was lower by 159–512 m than rebars without the protective coat placed in the same concrete. This can be attributed to zinc’s position against iron in the electrochemical series of metals. The potential drop was observed along with concrete hardening. It means that the most negative potentials were generally found for the last measurement series on day 25 from setting the specimens in concrete. The specimens G10-Cl–G12-Cl with galvanized rebars in concrete with chlorides were the exception. A reverse trend was observed for them.

### 4.3. Impedance Results for Reinforcing Steel in Concrete

**Figure 9:**
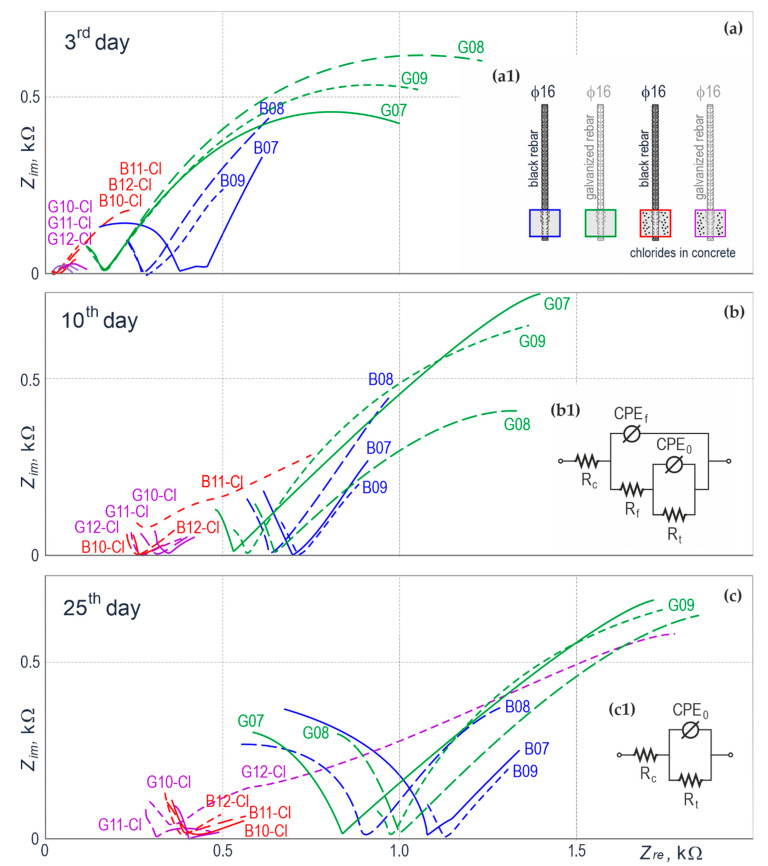
Impedance results on the Nyquist plot for ribbed rebars ϕ16 mm embedded in the concrete specimens: (**a**) the measurements on day 3 of concrete hardening in the specimens, (**a1**) 4 types of the elements tested with EIS, the colour of concrete paver outline corresponded to the colours of impedance spectra, (**b**) the measurements on day 10 after setting the specimens in concrete, (**b1**) equivalent electrical circuit for analysing the spectra of steel in concrete with chlorides, (**c**) the measurements on day 25 after setting the specimens in concrete, (**c1**) equivalent electrical circuit for analysing the spectra of steel in concrete without chlorides.

Figure 9 illustrates the results for impedance measurements taken for 12 specimens with ribbed rebars ϕ16 mm. Types of the test specimens are schematically presented in Figure 9(a1). The measurements with the EIS technique, similarly to the measurements of potential, were taken in 3 measurement series on days 3, 10, and 25 from the day of preparing the specimens. For better clarity of the presented results, different colours were attributed to the impedance spectra describing four types of the specimens: blue—the specimen with a black steel rebar in concrete without chlorides, green—the specimen with galvanized rebar in concrete without chlorides, red—the specimen with a black steel rebar in concrete with chlorides, violet—the specimen with the galvanized rebar in concrete with chlorides.

The impedance spectra in the complex *Z*_re_–*Z*_im_ plane are illustrated in Figure 9a–c. Their analysis shows that the impedance spectra for galvanized and black steel rebars in concrete with chlorides had considerably lower frequencies compared to the corresponding spectra for concrete without chlorides which indicated lower impedance of the described system. Low values of the impedance modulus in relation to flattened semi-circles (Figure 9a, specimens G10-Cl–G12-Cl) or shallow curves (Figure 9a–c, specimens B10-Cl–B12-Cl and Figure 9b,c, specimens G10-Cl–G12-Cl) within a low-frequency range can indicate the corrosion risk for steel in concrete. The impedance spectra for galvanized and black steel rebars in concrete without chlorides, on the other hand, show a few-fold higher impedance of the system. Two clear fragments can be observed for each spectrum. A curve or a small fragment of the flattened semi-circle was noticed on the left side of the characteristic inflection point of spectra, which was the closest to the horizontal axis of the real impedance. This part of the spectrum with high frequencies described the electrochemical properties of concrete and high-alkali liquid filling its pores. A considerably larger curve or a bigger fragment of the flattened semi-circle was noticed on the right side of the characteristic inflection point of spectra. This part with low frequencies was typical for the passivation of steel in concrete. A flattened shape of the semi-circle could indicate the initiation of gradual depassivation of metal.

The above qualitative analysis of experimentally determined 36 spectra was verified with the quantitative analysis based on adjusting theoretical spectra to corresponding electrical equivalent circuits illustrated in Figure 9(b1,c1). The spectra typical for galvanized and black steel rebars in concrete without chlorides were modelled using the modified Randles circuit (Figure 9(c1)), in which capacity was replaced with the constant phase element (CPE). Considering the spectra of rebars in concrete with chlorides, they were modelled using the circuit presented in Figure 9(b1) because the second time constant was noticed in the range of low frequencies. It should be mentioned that both equivalent electrical circuits were selected regarding only the analysis of low-frequency parts of spectra which were typical for electrochemical properties of metal in concrete. In both circuits (Figure 9(b1,c1)), the resistance *R*_c_ specified the impedance of the whole concrete, the resistance *R*_t_ specified the charge transfer resistance through the metal-pore solution interface, and the *CPE*_0_ characterized the pseudo-capacity of a double layer on steel. For the scheme illustrated in Figure 9(b1), the resistance *R*_f_ and the *CPE*_f_ specified the resistance and the pseudo-capacity of the forming layer of corrosion products of iron or zinc, which locally limited the access of oxygen to metal surface.

The detailed results for adjusting individual electrochemical parameters of both electrical equivalent circuits using the Simplex method are presented in Table A4, Appendix B. The charge transfer resistance *R*_t_, which was a key parameter, was used to calculate the density of corrosion current *i*_corr_ = *B*/*R*_t_ from the Stern-Geary equation. The parameter *B* was determined from the slope coefficients of linear sections of the polarization curves (anodic *b*_a_ and cathodic *b*_c_) obtained during the potentiodynamic tests conducted with the LPR technique described in point 4.4.

### 4.4. Potentiodynamic Results from Testing Polarization of Reinforcing Steel in Concrete

**Figure 10:**
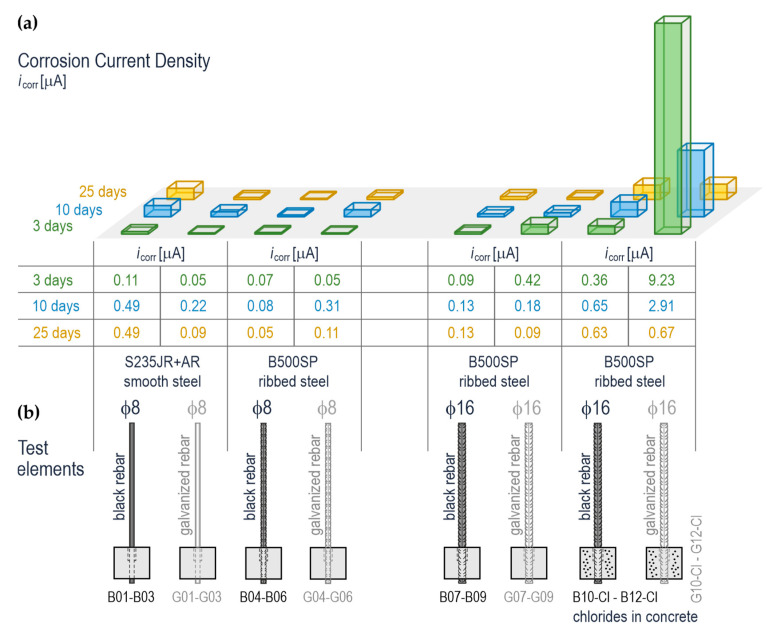
The results from electrochemical tests on reinforcing steel in concrete on days 3, 10, and 25 from setting the specimens in concrete: (**a**) corrosion current density *i*_corr_ determined with the LPR technique, (**b**) the test elements with galvanized and black reinforcing steel (smooth ϕ8 mm and ribbed ϕ8 and ϕ16 mm) in concrete with and without chlorides.

The potentiodynamic results from the LPR tests for the steel reinforcement in concrete performed after 3, 10, and 25 days from setting the specimens in concrete are shown in Figure 10. Contrary to the impedance tests specified in point 4.3, the direct current polarization measurements were taken for all 24 specimens as part of test stage 1—cf. Figure 3 A total of 72 polarization curves were obtained due to three measurement series taken within a few days at an interval of several days. By determining the polarization resistance *R*_p_ for each curve and the slope coefficient of a straight section of the anodic *b*_a_ and cathodic curves, the corrosion current density *i*_corr_ = B/R, where the parameter *B* = *b*_a_ *b*_c_/2.303 (*b*_a_ + *b*_c_), was calculated from the Stern-Geary equation. The densities of corrosion current averaged each time from three specimens are shown as a bar graph in Figure 10a. Different colours were attributed to individual measurement series for better clarity of the presented results. Additionally, the averaged densities of corrosion current *i*_corr_ expressed as [µA/cm^2^], are presented in the tabular form below the bar graph. Figure 10b shows sketches of all types of specimens tested with the LPR technique. They contain information on rebar diameter and steel grade, the presence or absence of the zinc coating and number and letter symbols.

The analysis of corrosion current densities presented that the following values *i*_corr_ = 0.05–0.13 µA/cm^2^ were obtained from all three measurement cycles for the specimens with black ribbed rebars ϕ8 mm and ϕ16 mm. This could indicate passivation. For the specimens made of smooth steel rebars ϕ8 mm, an increase in *i*_corr_ = 0.49 µA/cm^2^ was observed within two consecutive measurement cycles apart from the first one. This increase was not entirely explicable; however, it could suggest partial decomposition of the passive layer on metal. The expected increased values *i*_corr_ = 0.36–0.63 µA/cm^2^, indicating the development of corrosion, were found for unprotected ribbed reinforcing steel ϕ16 mm without zinc protection, which was set in concrete with aggressive chloride ions. In the case of the similar specimens but with the galvanized rebar, a dramatic rise in densities of the corrosion current up to *i*_corr_ = 9.23 µA/cm^2^ was noticed during the 1st cycle of measurements. This value was gradually falling in consecutive measurements to *i*_corr_ = 2.91 µA/cm^2^ on day 10, finally reaching the value *i*_corr_ = 0.67 µA/cm^2^ on day 25. It should be emphasized that very close range of corrosion current densities was obtained from the EIS tests, which had been conducted on a part of the specimens prior to the LPR tests. The observed effect of a rapid rise in the density of corrosion current can be attributed to the cumulative impact of chloride ions due to corrosion and high pH of pore solution, which caused partial dilution of the zinc coating at the initial stage of concrete setting. The values of corrosion current density for other specimens with galvanized rebars in concrete without chlorides ranged from 0.05 to 0.42 µA/cm^2^. These values usually did not exceed *i*_corr_ = 0.20 µA/cm^2^ indicating no signs of corrosion.

### 4.5. Results from Optical Measurements

**Figure 11:**
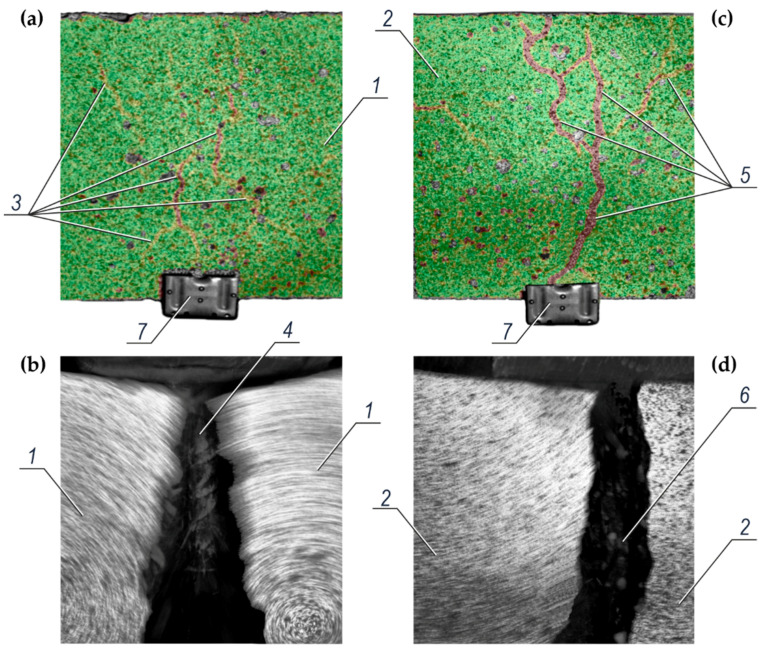
The analysis of the surface of concrete specimens with chlorides during the pull-out of ribbed rebars ϕ16 mm after 540 days from their preparation, which was conducted using the Aramis system: (**a**) surface of the specimen *1* (with a black steel rebar) and hardly visible dispersed brown grid *3* of displaced points sprayed with graphite paint, (**b**) the moment of rapid cracking *4* of concrete in the specimen *1* in the grid zone *3*, (**c**) surface of the specimen *2* (with the galvanized rebar) and very clear condensed brown grid *5* of displaced points, (**d**) the moment of rapid asymmetrical cracking *6* of concrete in the specimen *2* in the grid zone *5*; *7*—plate with permanent measuring points glued to the pulled out rebar.

The optical measurements with the Aramis camera system were additionally taken to provide new and detailed information on the strength tests. Their main objective was to record the displacements of points on the surface of the loaded tests element while pulling out the rebars from concrete causing the specimen failure. These measurements were used to catch the moment of fracture and crack formations in the test element, in which the reinforcement was anchored. Finding the connection between the force *N* acting on the rebar and its displacements s against the specimen was very important information used to determine the anchorage stiffness of rebar in concrete. Figure 11 presents the reference images of the surface of concrete specimens (containing chlorides) during the pull-out of ribbed rebars ϕ16 mm after 540 days from their preparation. A visible brown scattered grid *3* of displaced points is shown in Figure 11a. This image was captured during the pull-out of the black steel rebar. The pull-out of the galvanized rebar is presented in Figure 11c, where the very condensed brown grid *5* of the displaced points can be noticed. This grid changes into the asymmetrical crack *6* (bursting) of concrete *2* as presented in Figure 11d which illustrates the moment of rapid failure. Similarly, the moment of rapid cracking *4* of the specimen *1* is presented in Figure 11b. The crack symmetry can be related to the scattered, but almost centrally located, brown grid *3* of the displaced points that is shown in Figure 11a.

**Figure 12:**
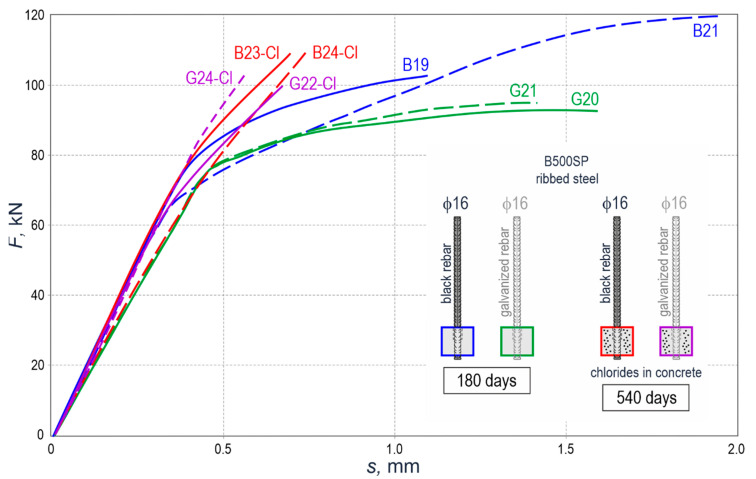
The relationship between the pull-out force *F* [kN] and the shift *s* [mm] during the pull-out of the galvanized and black steel rebar from concrete. This relationship, based on the Aramis system, was determined for rebars ϕ16 mm from steel grade B500SP—the tests performed after 180 and 540 days.

Figure 12 presents the results from measuring the rebar shift *s* [mm] from the concrete block caused by an increment of the pull-out force *F* [kN]. The measured shift was recorded using the Aramis camera system, whose software interacted with the control system for an increase in force of the strength machine. Due to the limited access to the Aramis system, the tests were reduced to galvanized and black steel ribbed rebars ϕ16 mm, embedded in concrete with and without chlorides. The elements without chlorides were tested after 180 days from the day of their preparation, and the elements containing chlorides were tested after 540 days.

The test results shown as curves in Figure 12 indicate the paths *F*(*s*) for the load range *F* = 0–60 kN were quasi-linear for each test element. A further increase in load, that is, for *F* > 60 kN, the elements containing chlorides B23-Cl, B24-Cl, G22-Cl, G24-Cl maintained their straight-line relationship *F*(*s*). On the other hand, the curves representing the elements (B19, B21, G20, G21) without chlorides in concrete had a clear drop in the slope *F*(*s*).

### 4.6. Results from Measuring the Force Pulling Out Rebars from Concrete

**Figure 13:**
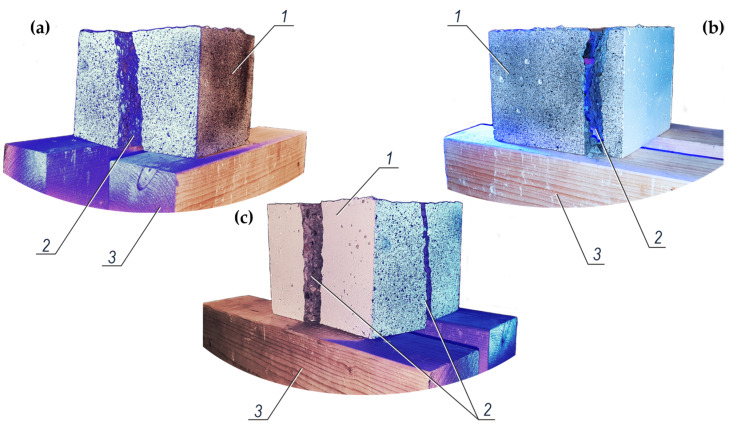
Images of typical failure of concrete specimens after pulling out the ribbed rebars of 16 mm in diameter: (**a**) uniform breaking of the specimen into two parts, (**b**) inclined breaking of the specimen into two parts, (**c**) breaking of the specimen into four parts; *1*—concrete specimen with the pulled-out reinforcement, *2*—cracked location in the specimen, *3*—wooden beams supporting the concrete specimens after pulling out the rebar.

All 48 test elements were damaged. Figure 13 shows the reference images of the damaged test elements after pulling out the ribbed rebars with a diameter of 16 mm. It can be seen that breaking of the concrete specimens was both symmetrical and asymmetrical into two (Figure 13a,b), and even four parts (Figure 13c).

**Figure 14:**
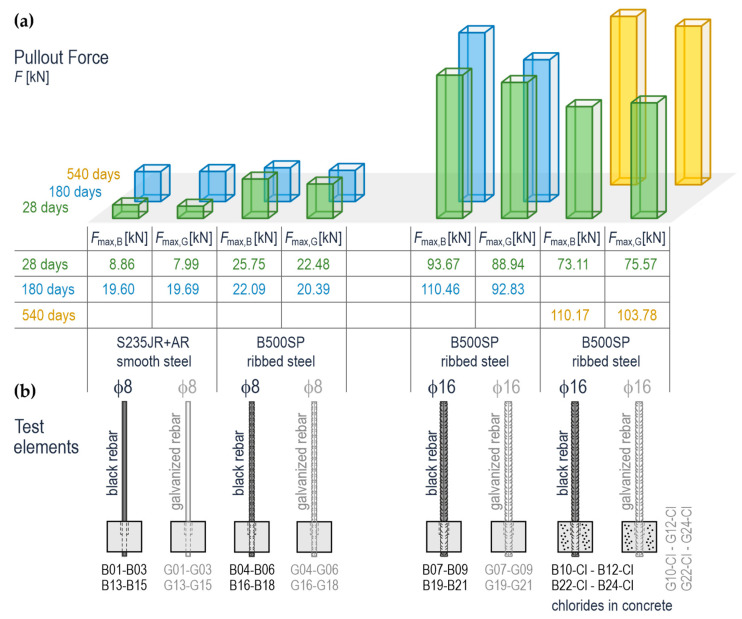
Mean values of the pull-out forces acting on galvanized and black steel (smooth ϕ8 mm and ribbed ϕ8 mm and ϕ16 mm) in the concrete specimens with and without chlorides after 28, 180, and 540 days from their preparation: (**a**) bar chart and corresponding values from the table, (**b**) the test elements; *F*_max,B_—the pull-out force acting on black steel rebars averaged from three specimens, *F*_max,G_—the pull-out force acting on galvanized rebars averaged from three specimens.

The bar graphs presented in Figure 14 compare values of the forces pulling out the rebars from concrete. The pull-out forces were averaged each time from three specimens. The notations *F*_max,G_ and *F*_max,B_ were used to differentiate the pull-out forces acting on the galvanized and black steel rebars, respectively. The values of the pull-out forces *F*_max_ determined for all 48 specimens are shown in Table A3, Appendix A. Different colours were attributed to determined forces to simplify the analysis of test results shown in Figure 14a: test stage 1—after 28 days from preparing the specimens (green), test stage 2—after 180 days (blue), and tests stage 3—after 540 days (yellow). Additionally, the sketches shown in Figure 14b representing the specimens with black steel reinforcement are in dark grey, and the ones with galvanized rebars are in light grey. To differentiate the results obtained for smooth steel (S235JR+AR) and ribbed steel, (B500SP), the grades and numbers of individual specimens are next to the specimen outlines.

The highest values of the pull-out forces (73.11–110.17 kN) were determined as expected for the ribbed rebars ϕ16 mm, and the lowest values (7.99–19.69 kN) for the smooth rebars ϕ8 mm. Except for the ribbed rebars ϕ8 mm, an increase in the pull-out force was noticed after 180 or 540 days. The greatest rise expressed in percentage was found for the smooth rebars ϕ8 mm. Considering the mean pull-out forces acting on the same specimens, the values obtained for galvanized rebars were generally lower compared to black steel rebars. Other results were obtained for smooth steel ϕ8 mm after 180 from setting the specimens in concrete and ribbed steel ϕ16 mm after 28 days from setting the specimens in concrete. A significant rise in the pull-out forces acting on the ribbed rebars ϕ16 mm after 540 days from preparing the specimens with chloride ions should be noted. These ions were aggressive for the reinforcement, so the specimens were very deteriorated due to corrosion.

**Figure 15:**
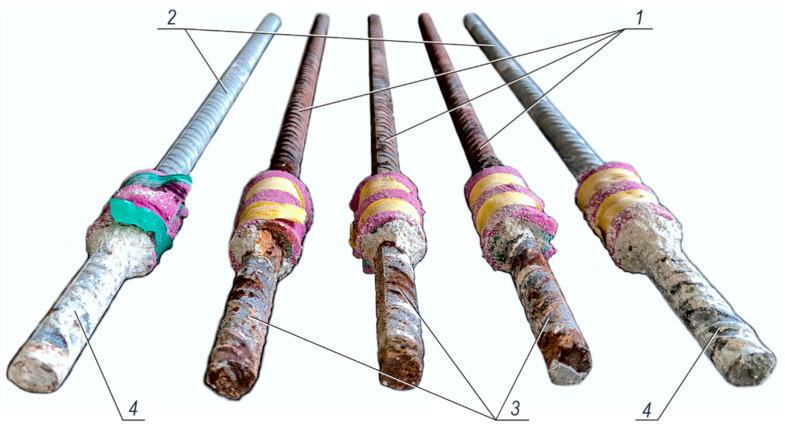
View of ribbed rebars ϕ16 mm made of steel grade B500SP pulled out from concrete after 540 days: *1*—black steel rebars, *2*—galvanized rebars, *3*—severely corroded fragments of black steel rebars in the area of their embedment in concrete, *4*—fragments of galvanized rebars embedded in concrete.

Figure 15 presents the images of five ribbed rebars ϕ16 mm after pulling out from the concrete block specimens. Black steel *1* and galvanized *2* rebars were embedded in concrete with chlorides of 3% concentration of the cement weight for the period of 540 days. It is important that the test elements with chlorides were stored within the last year in a closed chamber over a layer of water to provide the conditions which would intensify the development of reinforcement corrosion due to high relative humidity. Fragments of the rebar in the foreground were embedded in concrete specimens. Two edge rebars *4* were protected by zinc coating, on which the products of zinc corrosion in silver colour were not particularly distinguished. Three middle black steel rebars *3* had clear signs of plentifully deposited rusty products of iron corrosion.

## 5. Discussion

### 5.1. Causes of Failure of Test Elements

Two types of damage to the test elements were observed during the strength tests. The first type consisted in pulling out the anchored rebar from the concrete specimen without breaking the cover. In that case the concrete, in which the rebar was anchored, remained whole when the ultimate resistance of the element was exceeded. All elements reinforced with rebars of 8 mm in diameter were damaged in the above way. This failure occurred for the rebars made of both steel grades S235JR+AR and B500SP. Breaking of the concrete element, in which the rebar was anchored, was the second observed type of failure. It referred to all elements reinforced with rebars ϕ16 (B500SP). It should be emphasized that none of these elements were destroyed by the rupture of the anchored rebar.

The described types of failure of the elements are in line with the observations often described in the literature. Considering the smooth rebars, their failure was caused by pulling out the rebar from concrete, in which the rebar was anchored. The anchorage of ribbed rebars was also destroyed by pulling out the rebars from concrete providing that the reinforcement cover had adequate thickness [47]. In a situation like this, concrete was subjected to shear stress through ribs which was noticed for the rebars of 8 mm in diameter, where the cover thickness was 8.9ϕ. The cover failure occurred in the case of the rebars of 16 mm in diameter, where the cover thickness was 4.2ϕ.

To conduct a further analysis on resistance of the test elements, it is required to identify those specimens, for which the resistance failure appeared with the considerable plastic strain of the anchored reinforcement. As shown in point 2, six rebars of each diameter and steel grade were tested and the values of yielding force *F*_y_ were determined (Table A2, Appendix A). Assuming that *F*_y_ was a random variable with the standard distribution at the unknown standard deviation, then at the *α* significance level the *F*_y_ value was within the interval:

(1) $$p\left(F_{y,\mathrm{mean}}-t_{\alpha }\frac{s}{\sqrt{n}}<F_{y}<F_{y,\mathrm{mean}}+t_{\alpha }\frac{s}{\sqrt{n}}\right)=1-\alpha$$

where *F*_y,mean_—mean value of yielding force, *n*—number of specimens, *t*_α_—variable with the *t*—student distribution for *n* − 1 degrees of freedom, *s*—the value estimating the standard deviation for samples according to the following relationship

(2) $$s=\sqrt{\frac{1}{n-1}\overset{n}{\underset{i=1}{\sum }}{\left(F_{y,\mathrm{mean}}-F_{y,i}\right)}^{2}.}$$

The further analyses were performed using *α* = 0.02, for which *t*_α_ = 3.3649 at the *t*—student distribution for *n* − 1 degrees of freedom [48]. Based on the statistical analysis of the rebars presented in Table A3 (Appendix A), the values *F*_y_ for corresponding rebars were found to be within the following ranges with the probability of 1 − *α* = 0.98: ϕ8 (S235JR+AR) from 19.56 kN to 22.26 kN, ϕ8 (B500SP) from 21.91 kN to 28.75 kN and ϕ16 (B500SP) from 107.52 kN to 120.52 kN (Table 2).

In this context specifying a higher significance level, e.g., 0.05, would exclude the yielding forces that were experimentally determined, from the above ranges. Thus, a higher confidence level would lead to a contradiction. Knowing the upper and lower confidence limits for the forces *F*_y_, the direct cause of the anchorage failure could be specified. The failure caused by plastic strain occurred when the pull-out force *F* reached the value *F*_max_ higher than the lower confidence limit for *F*_y_, that is:

(3) $$\frac{F_{\max}}{\left(F_{y,\mathrm{mean}}-t_{\alpha }\frac{s}{\sqrt{n}}\right)}>1$$

If the sign of inequality in the relationship (3) was opposite, it means the element failure could be attributed to the exceeded critical stress of bond between rebars and concrete. However, if the following relationship would be satisfied

(4) $$\frac{F_{\max}}{\left(F_{y,\mathrm{mean}}+t_{\alpha }\frac{s}{\sqrt{n}}\right)}>1$$

then the failure of an element would be observed at stresses greater than the yield strength.

The values of the adequate quotients (3) and (4) are shown in Table A3, Appendix A. It follows that the relationship (4) was not satisfied for any element. Thus, the resistance of any element did not exceed the value corresponding to the force yielding the reinforcement. However, the relationship (3) was satisfied for some elements which means yielding of the reinforcement occurred. These values are indicated with the superscript letter (y) in Table 3.

### 5.2. Effect of Selected Parameters on Maximum Bond Stresses

Assuming that the uniform distribution of anchorage stress *f*_b_ was observed over the anchorage length *l*_b_, the stress values could be determined from the equilibrium state of the rebar

(5) $$f_{b}=\frac{F}{l_{b}u}$$

where *l*_b_ = 70 mm, *u*—rebar circumference. The maximum values of these stresses (*f*_b,max_) at the resistance loss are compared in Table 4. The superscript letter (y) written for the selected values means that the failure force acting on the test element was accompanied by the strain that is associated with yielding of the rebars.

To analyse the obtained results, the bond stress *f*_b,y,mean_ was determined in the first place which corresponded to the mean force *F*_y,mean_ yielding the reinforcement. The values *f*_b,y,mean_ were: 11.89 MPa for the rebars ϕ8 S235JR+AR, 14.40 MPa for the reinforcement ϕ8, B500SP, and 32.46 MPa for the rebars ϕ8, B500SP (Table 5).

It should be pointed out that the value *f*_b,y,mean_ for the rebar of 16 mm in diameter made of steel B500SP was more than twice the corresponding value for the same steel grade, but the diameter of 8 mm. This result was in line with the predictions because the value of adhesive stress *f*_b,y_ corresponding to stress *f*_y_ for the reinforcement could be expressed using the rearranged expression (5):

(6) $$f_{b,y}=\phi \frac{f_{y}}{4l_{b}}$$

Assuming that the stress *f*_b,y_, as *F*_y_, was the random variable, the corresponding confidence intervals at the significance level of 0.02 are additionally presented in Table 5.

The value *f*_b,y_ was the highest possible value of stress *f*_b_, which could be generated at the side wall of the rebar in concrete. It can be attributed to the mechanism of transmitting the force from the reinforcement to concrete. In the case of smooth rebars, where stresses *f*_b_ were caused by adhesion, the yielding force exerted on the rebar resulted in a rapid drop of its diameter, and consequently the adhesion force was eliminated due to the produced stresses perpendicular to the rebar axis. However, the force *F*_y_ initiated so significant deformation of anchored ribbed rebar that it could not be transmitted to concrete.

Table 6 contains the values expressed in percentage and determined from the equation:

(7) $$\frac{f_{b,\max}}{f_{b,y,\mathrm{mean}}}\cdot 100\%$$

**Figure 16:**
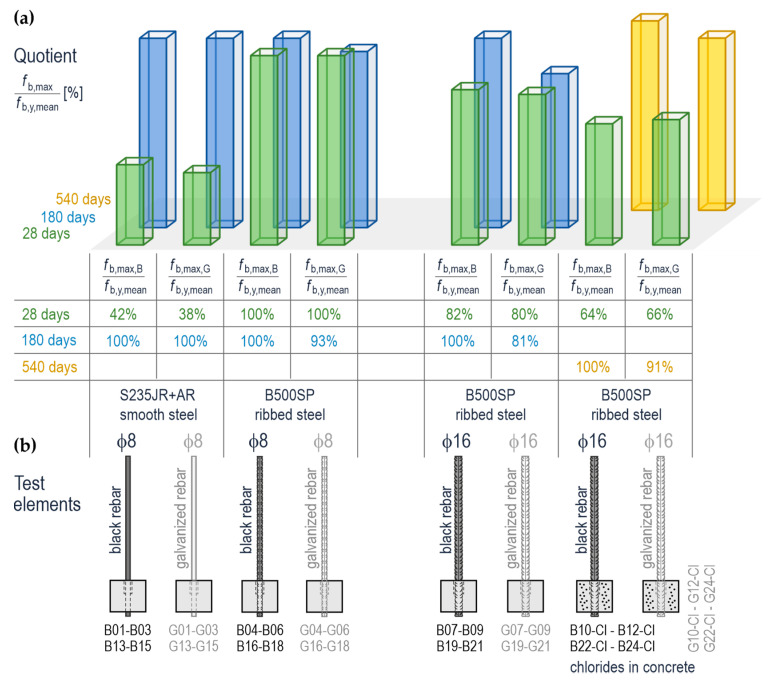
The percentage values determined from the relationship (7) based on the assumption that the parameter *f*_b_ of adhesive stresses is the random variable: (**a**) quotient *f*_b,max_/*f*_b,y,mean_ expressed as [%], (**b**) the test elements.

In this table *f*_b,max_ was considered the random variable. It means that the value determined from the Equation (7) takes 100% if f_b_ is within the adequate confidence level specified in Table 5. Graphical presentation of the results of calculations quotient (7) is illustrated in Figure 16. The impact of variable parameters of the test elements on the values *f*_b,max_ was determined from the analysis of the values presented in Figure 16.

Galvanization of smooth rebars at the beginning, that is, after 28 days from the day of their preparation, practically had no effect on the values *f*_b,max_ that could be found at the rebar surface. For the galvanized smooth rebars, the values *f*_b,max_ were lower by 4.2% compared to black steel smooth rebars. Similar observations were made for the ribbed rebars ϕ16 mm embedded in concrete without chlorides. The values *f*_b,max_ for galvanized rebars were lower by 2.5% than in the case of black steel rebars. Considering the rebars in concrete with chlorides, the reverse relationship was found. The values *f*_b,max_ were greater by 2.2% after 28 days. In the case of ribbed rebars of 8 mm in diameter, the galvanization had no effect on the value *f*_b,max_ after 28 days because the adhesive stresses in both cases reached the value *f*_b,y_.

The values *f*_b,max_ were increasing with the age of the test specimens which could be interpreted as a consequence of the increased strength of concrete. However, this increment was lower for the galvanized rebars with a diameter of 16 mm. The tests on the elements conducted after 180 days demonstrated that the value *f*_b,max_ for the galvanized elements was lower by 18.7% compared to the black steel elements. A similar effect was observed for the elements with chlorides tested after 540 days. In that case, the value *f*_b,max_ was lower by 9.1% which was not favourable to galvanized steel. A similar adverse effect of galvanization on the value *f*_b_ was found for the ribbed rebars of 8 mm in diameter that were tested after 180 days. In this case the values *f*_b_ for the galvanized rebars were lower by 9.3% compared to the black steel rebars. On the other hand, the value *f*_b_ determined for these rebars after 180 days was lower than after 28 days which was contrary to the expectations. It could indicate a serious drop in the resistance of anchorage of galvanized rebars over time. It should be mentioned that it was a single observation which could not be generalized.

The test results lead to the conclusions that chlorides in concrete had an impact on the values *f*_b,max_. Thus, *f*_b,max_ could be directly compared with the values determined after 28 days of concrete hardening. In the case of black steel rebars of 16 mm in diameter, chlorides reduced *f*_b,max_ by 18%. This effect was not so significant for the galvanized rebars as the values were reduced by 13.3%. The results determined after 540 days were likely to confirm the observations that the values f_b_ lower by 9.1% were found in the test elements with Cl. The value *f*_b,max_ was also affected by the process of galvanization.

### 5.3. Evaluation of Anchorage Stiffness of Rebars in Concrete

As described in point 4.5, the rebar shift s against the concrete specimen was recorded during the tests performed on the test elements containing the anchored rebars with a diameter of 16 mm and the measurements taken for the pull-out force *F*. These results are presented in the form of graphs in Figure 12. The relationships *F*(*s*) showed that these graphs were linear for a gradually increasing force *F* within the range of 0–60 kN. However, the shape of the graph *F*(*s*) was changing in the final stage of loading. For the elements without chlorides (B19, B21, G20, G21), a slope of the relationship *F(s*) was clearly decreasing. In the case of the elements containing chlorides (B23-Cl, B24-Cl, G22-Cl, G24-Cl), a slope of the curve with a quasi-linear shape was approximately constant throughout the whole loading cycle. Two phases were identified for each path of the relationship *F*(*s*) for the quantitative description of the above observations. The first phase included the loading range from 0 to 60 kN. The second phase was specified as the load contributing from 90% to 100% of the force *F*_max_. Then, the shift values *s* were read from Table 6 for typical loading levels. The values of stiffness *k* of the adhesive bond between the rebar and concrete were determined for both phases. They were considered as a quotient of an increment in loading Δ*F* and the corresponding increase in shift Δ*s*.

(8) $$k=\frac{\Delta F}{\Delta s}$$

**Figure 17:**
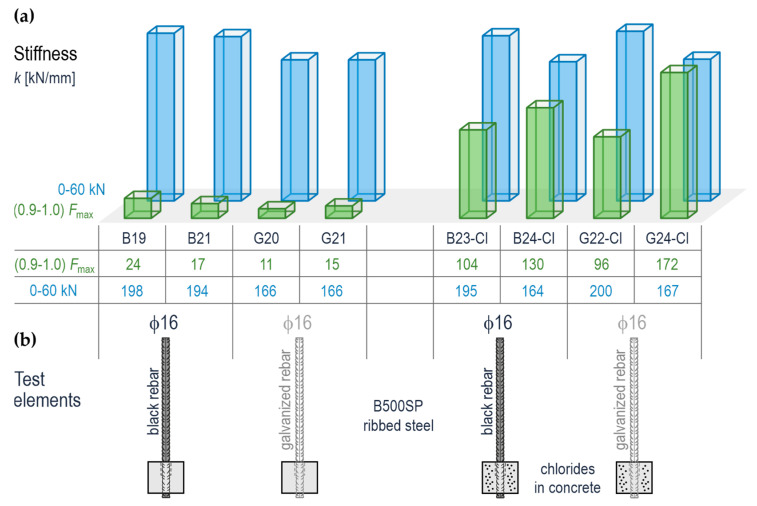
Anchorage stiffness of ribbed (galvanized and black steel) rebars ϕ16 mm in concrete (with and without chlorides) determined for two loading phases: phase 1—0–60 kN, phase 2—(0.9–1.0) *F*_max_: (**a**) stiffness of the test elements, (**b**) the test elements.

The calculated stiffness values are shown in Table 6 and illustrated in Figure 17. The bar chart in Figure 17a confirms the observations based on the analysis of the relationship *F*(*s*).

All the elements had significant stiffness values during the first phase of loading. The tests performed after 180 days demonstrated higher stiffness *k* during the first loading phase for the specimens with no chlorides and with black steel elements (197.7 kN/mm and 193.7 kN/mm) compared to the stiffness of galvanized rebars (166.34 kN/mm and 193.7 kN/mm). The stiffness of the elements was significantly reduced in the final loading phase. However, higher values were still noticed for the anchorage made of black steel (23.54 kN/mm and 17.32 kN/mm) compared to the anchorage of galvanized rebars (11.22 kN/mm and 14.57 kN/mm).

The elements with chlorides that were tested after 540 days demonstrated other stiffness values. Their initial stiffness within the loading range from 0 kN to 60 kN was 164.07–200.00 kN/m. In the final loading phase that covered from 90% to 100% *F*_max_, the stiffness slightly dropped to the level of 96.04–17.85 kN/m. The obtained results cannot be used to prove conclusively that galvanization of the rebars affects the stiffness of their anchorage in concrete after 540 days from the day of the preparation of these elements. However, the chlorides clearly demonstrated their impact on the stiffness in the final phase of loading. Chloride-induced corrosion of the reinforcement increased the anchorage stiffness in that phase by an order of magnitude.

According to the authors, the concrete age had no significant impact on the differences determined for observed stiffness values in the final phase of loading. The mean strength of concrete *f*_cm_ in the test elements at the age of 180 days was 47.70 MPa, and 50.16 MPa in the case of the test elements at the age of 540 days (Table A1, Appendix A). The modulus of concrete stiffness, estimated on the basis of the standard relationship [EC2],

(9) $$E_{\mathrm{cm}}\left(t\right)={\left(\frac{f_{cm}\left(t\right)}{f_{cm}}\right)}^{0.3}\cdot E_{cm}$$

increased from 35.15 GPa to 35.69 GPa (Table A1, Appendix A). This means that the products of chloride-induced corrosion, depositing in concrete pores in the immediate vicinity of the rebars, had a noticeable impact on the increased stiffness of the test bond.

## 6. Conclusions

The following conclusions can be drawn from testing the effect of the bond of hot-dip galvanized rebars made of steel grade B500SP and S235JR+AR to concrete and from the effects caused by chloride-induced corrosion of the reinforcement.

- The failure of all elements with ribbed rebars ϕ8 mm (B500SP) was accompanied by yielding of the reinforcement regardless of the age of the test elements. The presence of zinc coating on the rebars was not significant for the failure.
- Yielding of the elements reinforced with smooth rebars ϕ8 mm (S235JR+AR) was noticed only in the second stage of the tests, that is, after 180 days from their preparation. This observation also refers to the anchorage of galvanized and black steel rebars.
- Yielding of the reinforcement was not found in the case of failure of any test element containing galvanized ribbed rebars ϕ16 mm (B500SP). This fact could not be attributed to the age of the test elements or traces of products from zinc corrosion found after the pull-out of the rebars. This means that the zinc coating on the reinforcement inhibited the full use of mechanical properties of the rebars ϕ16 mm made of steel grade B500SP.
- In the case of the elements with the anchored black steel ribbed rebars ϕ16 mm (B500SP), yielding was observed only for the elements at the age of 180 and 540 days.
- Generally, zinc coating reduced the stiffness of anchorage of ribbed rebars ϕ16 mm (B500SP) This stiffness was reduced even by 15% when the pull-out force was within the range of 0–60 kN.
- However, the chloride-induced corrosion clearly demonstrated its impact on the anchorage stiffness in the final phase of loading. For the same content of chlorides in concrete, the development of corrosion of black steel was considerably more intensive and was related to greater volume of corrosion products on the rebar surface. The products from the corrosion of iron and zinc deposited in concrete pores and filled voids, which caused an increase in the anchorage stiffness in that phase by as much as one order of magnitude.

## Figures and Tables

**Table 1:** Maximum content of elements in the chemical composition of two tested steel grades B500SP and S235JR+AR.

Steel Grade	C	Mn	Si	P	S	Cu	N
B500SP	0.24%	1.65%	0.60%	0.06%	0.06%	0.85%	0.01%
S235JR+AR	0.17%	1.40%	–	0.045%	0.045%	–	0.009%

**Table 2:** The results from a statistical analysis for the forces *F_y_* yielding the test rebars.

Rebar Diameter (Steel Grade) (mm)	$F_{y,\mathrm{mean}}$ (KN)	$F_{y,\mathrm{mean}}-t_{\alpha }\frac{s}{\sqrt{n}}$ (KN)	$F_{y,\mathrm{mean}}+t_{\alpha }\frac{s}{\sqrt{n}}$ (KN)	*S* (kN)
ϕ8 (S235JR+AR)	20.92	19.56	22.26	0.98
ϕ8 (B500SP)	25.33	21.91	28.75	2.49
ϕ16 (B500SP)	114.20	107.52	120.52	4.86

**Table 3:** The superscript letter ^(y)^ specifying the mean pull-out forces *F*_max,B_ (the reinforcement without coating) and *F*_max,G_ (the galvanized reinforcement) accompanied by yielding of the rebars.

Time of Test (Day)	*F*_max,B_ (kN)	*F*_max,G_ (kN)	*F*_max,B_ (kN)	*F*_max,G_ (kN)	*F*_max,B_ (kN)	*F*_max,G_ (kN)	*F*_max,B_ (kN)	*F*_max,G_ (kN)
28	8.86	7.99	25.75 ^(y)^	22.48 ^(y)^	93.67	88.94	73.11	75.57
180	19.60 ^(y)^	19.69 ^(y)^	22.09 ^(y)^	20.39	110.46 ^(y)^	92.83		
540							110.17 ^(y)^	103.78
Rebar (steel grade)	ϕ8 (S235JR+AR) smooth steel		ϕ8 (B500SP) ribbed steel		ϕ16 (B500SP) ribbed steel		ϕ16 (B500SP) ribbed steel + Cl	

**Table 4:** Adhesive stresses *f*_b,max_ of reinforcing steel to concrete at the loss of the specimen resistance.

Time of Test (Day)	*f*_b,max,B_ (MPa)	*f*_b,max,G_ (MPa)	*f*_b,max,B_ (MPa)	*f*_b,max,G_ (MPa)	*f*_b,max,B_ (MPa)	*f*_b,max,G_ (MPa)	*f*_b,max,B_ (MPa)	*f*_b,max,G_ (MPa)
28	5.04	4.54	14.64 ^(y)^	12.78 ^(y)^	26.62	25.82	20.78	21.48
180	11.40 ^(y)^	19.69 ^(y)^	12.56 ^(y)^	11.59	31.39 ^(y)^	26.38		
540							31.30 ^(y)^	29.49
Rebar (steel grade)	ϕ8 (S235JR+AR) smooth steel		ϕ8 (B500SP) ribbed steel		ϕ16 (B500SP) ribbed steel		ϕ16 (B500SP) ribbed steel + Cl	

**Table 5:** The adhesive stress values *f*_b,y_ corresponding to the mean force *F*_y_ yielding the reinforcement and the confidence intervals at the significance level of 0.02 corresponding to these stresses.

Rebar Diameter (Steel Grade) (mm)	*f*_b,y,mean_ (MPa)	Minimum Value of Confidence Interval (MPa)	Maximum Value of Confidence Interval (MPa)
ϕ8 (S235JR+AR)	11.89	11.12	12.65
ϕ8 (B500SP)	14.40	12.45	16.34
ϕ16 (B500SP)	32.46	30.56	34.25

**Table 6:** Typical parameters determined from the graphs in Figure 12, based on the analysis of the relationship between the pull-out force *F* [kN] and shifts *s* [mm] of rebars: 0.9 *F*_max_ and *F*_max_—90% and 100% respectively, of the maximum pull-out force, *s* (60 kN), *s* (0.9 *F*_max_) and *s* (*F*_max_)—shift *s* of the rebar from the concrete specimen for the force of 60 kN, 90% *F*_max_ and *F*_max_, *k* (0–60 kN) and *k* [(0.9–1.0) *F*_max_]—stiffness *k* of the adhesive bond between the rebar and concrete for the force values within the range of 0–60 kN and 90–100% of the force *F*_max_.

Specimens	0.9 *F*_max_ (kN)	*F*_max_ (kN)	*s* (60 kN) (mm)	*s* (0.9 *F*_max_) (mm)	*s* (*F*_max_) (mm)	*k* (0–60 kN) (kN/mm)	*k* (0.9–1.0) *F*_max_ (kN/mm)
B19	92.12	102.35	0.30	0.64	1.07	197.70	23.54
B21	107.36	119.29	0.31	1.27	1.96	193.70	17.32
G20	83.68	92.97	0.36	0.66	1.49	166.32	11.22
G21	85.43	94.92	0.36	0.72	1.37	166.34	14.57
B23-Cl	97.97	108.86	0.31	0.59	0.69	194.72	104.23
B24-Cl	100.27	111.41	0.37	0.66	0.75	164.07	130.32
G22-Cl	89.82	99.8	0.30	0.57	0.67	200.00	96.04
G24-Cl	94.17	104.63	0.37	0.49	0.56	167.07	171.85

## Data Availability

The data presented in this study are available on request from the corresponding author.

## Appendix A

**Table A1:** Compressive strength of concrete in the test elements determined by testing cylindrical specimens with a diameter of 150 mm and a height of 300 mm after 28, 180, and 540 days from setting them in concrete.

Time (Day)	*f*_ci_ (MPa)	*f*_cm_ (MPa)	*E*_cm_ (GPa)
28	31.89		
28	43.09		
28	40.92		
28	33.95	38.22	32.9
28	33.05		
28	46.42		
180	58.49		
180	40.99		
180	41.75		
180	51.85	47.70	39.7
180	53.00		
180	40.14		
540	54.64		
540	55.03		
540	52.43		
540	41.54	50.16	42.2
540	42.24		
540	55.06		

**Table A2:** Strength characteristic for all types of the test rebars used in the tests, determined from the graphs of “stress σ–strain ε” illustrated in Figure 2: *F*_y_—the force corresponding to yield strength of steel *f*_y_, *F*_y,mean_—the force corresponding to yield strength of steel *f*_y_ averaged from six values *ε*_u_—steel strains at the maximum force, *F*_t_—the force corresponding to tensile strength of steel *f*_t._

Rebar Diameter (Steel Grade)	Type of Steel Rebar	*F*_y_ (kN)	*F*_y,mean_ (kN)	*ε*_u_ (%)	*F*_t_ (kN)	*F*_t_/*F*_y_
ϕ8 (S235JR+AR)	black smooth	20.02		15	24.24	1.21
ϕ8 (S235JR+AR)	black smooth	19.87		15	23.89	1.20
ϕ8 (S235JR+AR)	black smooth after pull-out	20.22		14	24.45	1.21
ϕ8 (S235JR+AR)	galvanized smooth	21.58	20.92	18	25.35	1.17
ϕ8 (S235JR+AR)	galvanized smooth	22.08		13	25.85	1.17
ϕ8 (S235JR+AR)	galvanized smooth after pull-out	21.73		17	26.41	1.22
ϕ8 (B500SP)	black ribbed	23.89		18	29.98	1.25
ϕ8 (B500SP)	black ribbed	23.99		19	29.78	1.24
ϕ8 (B500SP)	black ribbed after pull-out	25.70		8	31.29	1.22
ϕ8 (B500SP)	galvanized ribbed	29.02	25.33	15	32.95	1.14
ϕ8 (B500SP)	galvanized ribbed	22.18		12	26.21	1.18
ϕ8 (B500SP)	galvanized ribbed after pull-out	27.21		17	31.24	1.15
ϕ16 (B500SP)	black ribbed	116.78		11	139.49	1.19
ϕ16 (B500SP)	black ribbed	116.98		11	139.90	1.20
ϕ16 (B500SP)	black ribbed after pull-out	115.17		10	138.29	1.20
ϕ16 (B500SP)	galvanized ribbed	107.74	114.20	4	117.38	1.09
ϕ16 (B500SP)	galvanized ribbed	108.74		6	122.21	1.12
ϕ16 (B500SP)	galvanized ribbed after pull-out	119.80		11	141.10	1.18

**Table A3:** The values of the pull-out force *F* acting on galvanized and black steel rebars in concrete with and without chlorides on day 28, 180, and 540 from the day of their setting in concrete. *F*_y,mean_—the force corresponding to yield strength of steel *f*_y_ averaged from six values. *n*—number of the specimens (*n* = 6), *t*_α_—variable with the t-student distribution for *n* − 1 degrees of freedom, *s*—the value estimating the standard deviation for samples according to the relationship (2), *F*_B_ and *F*_G_—the value of the pull-out force acting on galvanized and black steel rebars averaged from three measurements.

Time (Day)	Specimen No.	Rebar Diameter (Steel Grade) (mm)	*F*_max_ (kN)	$\frac{F}{\left(F_{y,\mathrm{mean}}-t_{\alpha }\frac{s}{\sqrt{n}}\right)}$	$\frac{F}{\left(F_{y,\mathrm{mean}}+t_{\alpha }\frac{s}{\sqrt{n}}\right)}$	*F*_max,B_ *F*_max,G_ (kN)
28	B01	ϕ8 (S235JR+AR)	8.47	0.43	0.38	
28	B02	ϕ8 (S235JR+AR)	6.11	0.31	0.27	8.86
28	B03	ϕ8 (S235JR+AR)	12.01	0.61	0.54	
28	G01	ϕ8 (S235JR+AR)	7.67	0.39	0.34	
28	G02	ϕ8 (S235JR+AR)	7.51	0.38	0.34	7.99
28	G03	ϕ8 (S235JR+AR)	8.79	0.45	0.39	
28	B04	ϕ8 (B500SP)	26.75	1.22	0.93	
28	B05	ϕ8 (B500SP)	25.99	1.19	0.90	25.75
28	B06	ϕ8 (B500SP)	24.51	1.12	0.85	
28	G04	ϕ8 (B500SP)	22.61	1.03	0.79	
28	G05	ϕ8 (B500SP)	21.56	0.98	0.75	22.48
28	G06	ϕ8 (B500SP)	23.27	1.06	0.81	
28	B07	ϕ16 (B500SP)	85.13	0.79	0.70	
28	B08	ϕ16 (B500SP)	98.54	0.92	0.82	93.67
28	B09	ϕ16 (B500SP)	97.39	0.91	0.81	
28	G07	ϕ16 (B500SP)	87.44	0.81	0.72	
28	G08	ϕ16 (B500SP)	90.88	0.85	0.75	88.94
28	G09	ϕ16 (B500SP)	88.49	0.82	0.73	
28	B10-Cl	ϕ16 (B500SP)	67.33	0.63	0.56	
28	B11-Cl	ϕ16 (B500SP)	73.08	0.68	0.60	73.11
28	B12-Cl	ϕ16 (B500SP)	78.91	0.73	0.65	
28	G10-Cl	ϕ16 (B500SP)	73.27	0.68	0.61	
28	G11-Cl	ϕ16 (B500SP)	75.26	0.70	0.62	75.57
28	G12-Cl	ϕ16 (B500SP)	78.18	0.73	0.65	
180	B13	ϕ8 (S235JR+AR)	20.25	1.04	0.91	
180	B14	ϕ8 (S235JR+AR)	19.03	0.97	0.85	19.60
180	B15	ϕ8 (S235JR+AR)	19.51	1.00	0.88	
180	G13	ϕ8 (S235JR+AR)	18.81	0.96	0.84	
180	G14	ϕ8 (S235JR+AR)	20.11	1.03	0.90	19.69
180	G15	ϕ8 (S235JR+AR)	20.15	1.03	0.90	
180	B16	ϕ8 (B500SP)	21.41	0.98	0.74	
180	B17	ϕ8 (B500SP)	22.38	1.02	0.78	22.09
180	B18	ϕ8 (B500SP)	22.48	1.03	0.78	
180	G16	ϕ8 (B500SP)	20.39	0.93	0.71	
180	G17	ϕ8 (B500SP)	19.74	0.90	0.69	20.39
180	G18	ϕ8 (B500SP)	21.04	0.96	0.73	
180	B19	ϕ16 (B500SP)	102.35	0.95	0.85	
180	B20	ϕ16 (B500SP)	109.74	1.02	0.91	110.46
180	B21	ϕ16 (B500SP)	119.29	1.11	0.99	
180	G19	ϕ16 (B500SP)	90.61	0.84	0.75	
180	G20	ϕ16 (B500SP)	92.97	0.86	0.77	92.83
180	G21	ϕ16 (B500SP)	94.92	0.88	0.79	
540	B22-Cl	ϕ16 (B500SP)	109.87	1.02	0.91	
540	B23-Cl	ϕ16 (B500SP)	108.85	1.01	0.90	110.07
540	B24-Cl	ϕ16 (B500SP)	111.48	1.04	0.92	
540	G22-Cl	ϕ16 (B500SP)	99.80	0.93	0.83	
540	G23-Cl	ϕ16 (B500SP)	106.91	0.99	0.88	103.78
540	G24-Cl	ϕ16 (B500SP)	104.63	0.97	0.87	

## Appendix B

**Table A4:** Comparison of the results from analysing the impedance spectra of galvanized and black steel rebars ϕ 16 mm, made of steel grade B500SP, embedded in concrete with and without chlorides. The tests were conducted on days 1, 7, and 28 after setting the specimens in concrete.

Specimen No.	Δ*t* (Day)	*E*_Ag|AgCl_ (V)	*R*_c_ (Ω)	*R*_f_ (Ω)	*CPE*_f_		*R*_t_ (Ω)	CPE_0_		*i_corr_*
					*Y*_f_ (Fs^α^^− 1^)	*α*_f_		*Y*_0_ (Fs^α^^−1^)	*α*_0_	
B07	3	−0.097	444				2.50 × 10^4^	1.18 × 10^−2^	0.715	0.01
B08	3	−0.173	276				1.07 × 10^4^	4.05 × 10^−3^	0.587	0.02
B09	3	−0.175	290				8.68 × 10^4^	6.54 × 10^−3^	0.524	0.00
G07	3	−0.333	171				1.75 × 10^3^	1.70 × 10^−3^	0.655	0.24
G08	3	−0.292	170				2.32 × 10^3^	1.35 × 10^−3^	0.672	0.21
G09	3	−0.302	174				2.01 × 10^3^	1.46 × 10^−3^	0.664	0.23
B10-Cl	3	−0.354	26	28	9.34 × 10^−3^	0.551	2.30 × 10^3^	4.62 × 10^−2^	0.625	0.06
B11-Cl	3	−0.378	24	1	7.86 × 10	0.029	1.13 × 10^3^	6.86 × 10^−3^	0.588	0.24
B12-Cl	3	−0.379	23	11	1.19 × 10^−2^	0.526	8.88 × 10^3^	2.32 × 10^−2^	0.612	0.01
G10-Cl	3	−0.857	27	3	5.28 × 10^−1^	0.099	3.41 × 10^1^	2.23 × 10^−2^	0.461	9.95
G11-Cl	3	−0.880	36	1	5.88 × 10^−3^	0.671	3.44 × 10^1^	1.43 × 10^−2^	0.527	9.48
G12-Cl	3	−0.872	29	33	1.48 × 10^−2^	0.619	3.28 × 10^1^	1.78 × 10^−2^	0.521	9.90
B07	10	−0.166	701				9.71 × 10^3^	6.63 × 10^−3^	0.572	0.02
B08	10	−0.238	646				6.42 × 10^3^	3.70 × 10^−3^	0.634	0.03
B09	10	−0.207	596				9.32 × 10^3^	3.70 × 10^−3^	0.598	0.02
G07	10	−0.629	538				5.06 × 10^3^	1.69 × 10^−3^	0.566	0.06
G08	10	−0.752	663				1.68 × 10^3^	1.80 × 10^−3^	0.602	0.23
G09	10	−0.713	648				4.45 × 10^3^	1.89 × 10^−3^	0.614	0.09
B10-Cl	10	−0.430	257	127	1.22 × 10^−2^	0.278	4.43 × 10^2^	1.53 × 10^−1^	0.962	0.58
B11-Cl	10	−0.853	229	4949	3.04 × 10^−4^	0.653	2.01 × 10^3^	4.63 × 10^−5^	0.460	0.38
B12-Cl	10	−0.427	130	151	1.01 × 10^−3^	0.049	1.06 × 10^3^	1.80 × 10^−2^	0.406	0.24
G10-Cl	10	−0.686	325	89	3.71 × 10^−2^	0.256	8.62 × 10^1^	1.65 × 10^−1^	0.873	2.85
G11-Cl	10	−0.778	21	2463	6.03 × 10^−2^	0.328	1.10 × 10^2^	5.43 × 10^−4^	0.029	2.46
G12-Cl	10	−0.830	269	14	1.60 × 10^−1^	0.744	5.91 × 10^1^	1.45 × 10^−2^	0.504	3.29
B07	25	−0.215	1066				1.40 × 10^5^	5.29 × 10^−3^	0.456	0.01
B08	25	−0.259	901				4.79 × 10^+3^	3.76 × 10^−3^	0.564	0.04
B09	25	−0.238	1237				1.81 × 10^4^	5.81 × 10^−3^	0.496	0.01
G07	25	−0.684	838				3.88 × 10^3^	1.58 × 10^−3^	0.541	0.06
G08	25	−0.723	1011				3.28 × 10^3^	1.67 × 10^−3^	0.577	0.07
G09	25	−0.617	997				3.08 × 10^3^	1.97 × 10^−3^	0.507	0.08
B10-Cl	25	−0.388	400	6261	1.24 × 10^−2^	0.276	9.73 × 10^1^	7.80 × 10^−5^	0.776	1.78
B11-Cl	25	−0.421	372	323	5.34 × 10^−3^	0.325	9.50 × 10^1^	4.15 × 10^−5^	1.000	1.87
B12-Cl	25	−0.396	391	12	4.00 × 10^−4^	0.746	3.89 × 10^2^	1.92 × 10^−2^	0.506	0.40
G10-Cl	25	−0.644	402	94	4.73 × 10^−3^	0.402	1.79 × 10^2^	3.20 × 10^−3^	0.000	1.38
G11-Cl	25	−0.745	229	72750	6.30 × 10^−3^	0.064	1.88 × 10^2^	1.19 × 10^−4^	0.833	1.23
G12-Cl	25	−0.429	304	1065	2.05 × 10^−4^	0.397	5.13 × 10^2^	6.29 × 10^−4^	0.563	0.52
